# Direct numerical simulation of the turbulent flow around a Flettner rotor

**DOI:** 10.1038/s41598-024-53194-x

**Published:** 2024-02-06

**Authors:** Daniele Massaro, Martin Karp, Niclas Jansson, Stefano Markidis, Philipp Schlatter

**Affiliations:** 1 SimEx/FLOW, Engineering Mechanics, KTH Royal Institute of Technology, Stockholm, Sweden; 2 Division of Computational Science and Technology, EECS, KTH Royal Institute of Technology, Stockholm, Sweden; 3 PDC Centre for High Performance Computing, EECS, KTH Royal Institute of Technology, Stockholm, Sweden; 4 Institute of Fluid Mechanics (LSTM), Friedrich–Alexander–Universität (FAU), Erlangen–Nürnberg, Germany

**Keywords:** Fluid dynamics, Aerospace engineering

## Abstract

The three-dimensional turbulent flow around a Flettner rotor, i.e. an engine-driven rotating cylinder in an atmospheric boundary layer, is studied via direct numerical simulations (DNS) for three different rotation speeds ($\alpha$). This technology offers a sustainable alternative mainly for marine propulsion, underscoring the critical importance of comprehending the characteristics of such flow. In this study, we evaluate the aerodynamic loads produced by the rotor of height *h*, with a specific focus on the changes in lift and drag force along the vertical axis of the cylinder. Correspondingly, we observe that vortex shedding is inhibited at the highest $\alpha$ values investigated. However, in the case of intermediate $\alpha$, vortices continue to be shed in the upper section of the cylinder ($y/h>0.3$). As the cylinder begins to rotate, a large-scale motion becomes apparent on the high-pressure side, close to the bottom wall. We offer both a qualitative and quantitative description of this motion, outlining its impact on the wake deflection. This finding is significant as it influences the rotor wake to an extent of approximately one hundred diameters downstream. In practical applications, this phenomenon could influence the performance of subsequent boats and have an impact on the cylinder drag, affecting its fuel consumption. This fundamental study, which investigates a limited yet significant (for DNS) Reynolds number and explores various spinning ratios, provides valuable insights into the complex flow around a Flettner rotor. The simulations were performed using a modern GPU-based spectral element method, leveraging the power of modern supercomputers towards fundamental engineering problems.

## Introduction

Over the past few decades, the pursuit of solutions to enhance transportation efficiency and mitigate fuel consumption has been a central focus for researchers. In the early twentieth century, when the climate crisis was far from being in the spotlight, the German inventor Anton Flettner designed a new kind of rotor sail^1^. The Flettner rotor consists of a rotating, engine-driven cylinder which extends vertically and exploits the Magnus effect: when the lateral wind is blowing, a component of the aerodynamic force in the thrust direction is generated. In this way, the ship is driven forward by harvesting the free-of-charge wind energy. It obtains a reduction in fuel consumption of up to 20%, in a sector such as maritime transport which constitutes a large portion of global shipping, responsible for more than 2% of all carbon emissions^2^. In working conditions, the Flettner rotor has a diameter and height of 3–5 and 20–30 meters, respectively. It stands on the ship deck in the turbulent atmospheric boundary layer, which has a thickness of around several tens of meters. The height of the deck is not considered in this exploratory study. The spinning ratio $\alpha =U_{sp}/U_{cl}$, usually varies between $1<\alpha <5$, where $U_{sp}$ is the spinning velocity of the cylinder and $U_{cl}$ is the centre-line velocity further away from the wall. Such canonical a configuration has been barely studied in the past. Indeed, mostly homogeneous and parallel inflow conditions to the rotating cylinder were considered, ignoring the fact that a Flettner rotor operates in an atmospheric shear layer.

Spanning almost one century of literature, several studies calculated integral quantities and wake deficits in the three-dimensional flow past a rotor^3–7^. In particular, at relatively high Reynolds numbers, the main focus was the measurements of the aerodynamic forces, i.e. lift and drag^8–10^. Several discrepancies have arisen during the years and there are various reasons such as the absence of a unified benchmark, which has led to a variety of different aspect ratios and Reynolds numbers being considered, as well as limitations in the numerical approach. The numerical simulations currently available typically integrate the Reynolds averaged Navier–Stokes (RANS) equations, where the choice of the closure model plays a crucial role. In recent experimental campaigns^11,12^, it has been concluded that, for a Flettner rotor, only the lift coefficient is affected by the Reynolds number when the spinning ratio is significantly high, e.g. $\alpha =2.5$. Conversely, for $1<\alpha <2.5$, the drag coefficient is affected by the Reynolds number. More complex configurations, in tandem or with a flap at the free-end surface, have also been considered^13^. However, all of these studies focus on a homogeneous parallel inflow. High-fidelity numerical or experimental studies that encompass various levels of complexity, such as non-homogeneous inflow or the wall-cylinder interaction, are lacking. A recent study by Cao et al.^14^ explored a setup similar to ours, involving a square cylinder in a thick boundary layer, without accounting for the rotational effects.

Regarding the study of rotating cylinders with a homogeneous inflow, several studies can be found in the literature^15–17^. Yet, the flow still presents unresolved issues and open questions. The common experience suggests that increasing the spinning rate leads to a significant increase in the lift coefficient. The underlying mechanism is the well-known Magnus effect^18^ and finds an intuitive explanation in potential theory^19^, which predicts the pressure imbalance generated by the spinning motion. The resulting force acts orthogonal to the inflow direction. Even so, to what extent can we observe such an increase? Prandtl^20^ determined that the maximum lift generated by a spinning cylinder in a uniform flow is constrained to an asymptotic value. Subsequent studies delved into this matter, and comprehensive summaries are available in the literature^15,21^. Nevertheless, there is ongoing debate regarding the asymptotic maximum lift generated by a rotating cylinder, as predicted by Prandtl. Many studies have noted that at high rotation rates, the lift can exceed the maximum limit predicted by Prandtl^15,22–24^. A second crucial question is the vortex shedding suppression due to the cylinder rotation. The experiments and simulations performed by Refs.^25–27^ agree that beyond a critical rotation rate, only one vortex is shed. This critical $\alpha$ is almost independent of Re and is approximately 2. For $\alpha >2$, the Kármán activity in the wake deteriorates and the vortex shedding is suppressed. Differently, Chen et al.^24^ observed that more than one vortex is shed for $\alpha =2$ and $\alpha =3.25$ at $\textrm{Re}_D=200$ (based on the homogeneous inflow velocity and the diameter *D*), with the vortices shed at later times being much weaker. However, their finding was substantially erroneous because they did not let the flow evolve for enough time (see Figure 2 in Mittal and Kumar^15^). Increasing the Reynolds number even further from $\textrm{Re}_D=400$ to $\textrm{Re}_D=1000$, Padrino and Joseph^28^ confirmed the results by Mittal and Kumar^15^. In a similar way, other studies for $\alpha =1$^29,30^ and $\alpha \le 2$, at very high Reynolds number^31^, describe the physics of the flow in agreement with the conclusions drawn by Mittal and Kumar^15^.

One additional phenomenon is worthwhile mentioning, with potentially significant implications for lift production, namely the so-called *inverse Magnus effect*. Under certain conditions, at comparably high Reynolds numbers and within a specific range of rotation speeds, the lift direction may change by 180^∘^^32^. The exact reasons for this behaviour and detailed analysis of the parameter ranges are yet unclear in the literature. However, the reasons are thought to be related to the drag crisis of the cylinder flow when the boundary layers become turbulent. The delayed/promoted transition to turbulence on the two sides of the rotating cylinder then leads to an inverse effect of the pressure-force distribution. Given the high Reynolds number ($\textrm{Re}_D \approx$ 100,000–200,000) required, and the necessity to fully resolve the boundary layers of the cylinder, no detailed study is present in the literature, apart from the experimental flow around a sphere^32^.

As outlined by the above overview, the previous studies either consider fundamental configurations, i.e. rotating cylinders at relatively low Reynolds number and with homogeneous inflow, or either perform low-fidelity simulations, heavily dependent on the modelling choice. Differently, we aim to investigate the flow around a cylinder in a thick boundary layer at a moderately high Reynolds number. This constitutes a good model for the Flettner rotor in the atmospheric boundary layer. The wake characterisation and dynamic load variation on the cylinder have crucial consequences on the shipping efficiency and the structural stability of the ship itself. To the authors’ knowledge, this study is the first-ever, three-dimensional direct numerical simulation (DNS) of a rotating cylinder in a wall-bounded shear flow.

The manuscript is organised as follows: first, we outline the flow configuration, and then we present and discuss the main results. In Sects. 4 and 5, we provide the conclusions and methodology, respectively.

**Figure 1:**
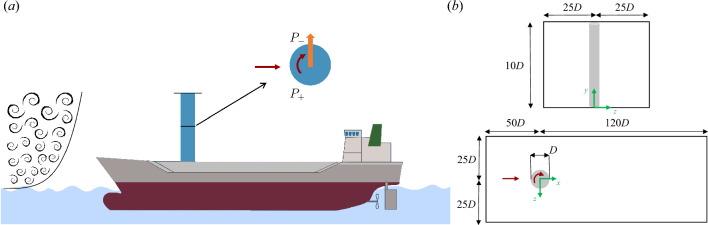
(**a**) Illustration of a ship with a Flettner rotor (in blue). The (turbulent) atmospheric boundary layer and the generated lateral force are also highlighted. Low- and high-pressure sides are indicated with $P_{-}$ and $P_{+}$, respectively. The figure provides an overview of the simulation setup. However, it remains just an illustration as many aspects are not considered in this study, e.g. the boundary layer-ocean interaction and the height of the deck. (**b**) Sketch of the simulation setup, with the frame of reference (in green) and the dimensions of the domain. The red arrows indicate the inflow and spinning velocities. The coordinate system is such that the streamwise direction is *x*, the vertical direction *y* and the spanwise *z*. Note that the sketch of the computational domain is not to scale.

## Flow configuration

In the current study, we perform a direct numerical simulation (DNS) of a Flettner rotor in a turbulent boundary layer. To model the atmospheric boundary at an affordable cost, a turbulent open channel flow is simulated, i.e. a shear flow, wall-bounded on one side^33^. The three-dimensional cylinder is located at the origin of our domain and rotates clockwise w.r.t. the *y* direction (viewed from the top in Fig. 1a). The reference system has the *y*-axis aligned with the cylinder vertical axis, and the *x* and *z*-axes represent the streamwise and spanwise directions, respectively. A sketch is provided in Fig. 1. The Cartesian mesh uses a block-structured configuration with approximately 6 billion unique grid points, with the presence of 12 million spectral elements, with a polynomial order $N=7$. Further details about the numerical methodology are provided in Sect. 5. To prevent undesired boundary effects, we carefully designed the domain. The chosen sizes are significantly larger than any previous DNS of rotating cylinders or bluff bodies in shear flows: $(-50D, 120D)$ in *x*, $(-25D, 25D)$ in *z*, and (0, 10*D*) in *y*, where *x*, *z*, and *y* represent the streamwise, spanwise, and vertical directions, respectively.

The flow dynamics is fully described by three non-dimensional parameters:

- The Reynolds number, based on the centre-line velocity, $U_{cl}$, and the height, *h*, of the open channel: $\textrm{Re}_{cl} = \frac{U_{cl}h}{\nu }$, where $\nu$ represents the kinematic viscosity of the fluid,
- The aspect ratio between the height *h* and the cylinder diameter *D* ($\gamma =h/D$),
- The rotation, or spinning, ratio between the centre-line, $U_{cl}$, and the spinning cylinder velocity, $U_{sp}$ ($\alpha =U_{sp}/U_{cl}$).

In our setup, the aspect ratio is $\gamma =10$ and the Reynolds number is $\textrm{Re}_{cl}=30,000$, corresponding to a Reynolds number based on the cylinder diameter of $\textrm{Re}_{D}=U_{cl} D/\nu =3000$. Three different spinning ratios are considered $\alpha =0, 1.5$ and 3. Within the limits of DNS (where all the scales are simulated and no turbulence model is introduced), we aim to reproduce a realistic configuration. Indeed, in operative conditions, the Flettner rotor has an aspect ratio $\gamma \approx$ 7–12 and spinning ratio $1<\alpha <5$, although the Reynolds number is higher ($\textrm{Re}_{cl} \ge 10^{5}$).

The main effect that is not included in the present study is the impact of the top boundary of the cylinder, and the vortices potentially arising at that interface. Studying a finite rotating cylinder in a boundary layer is certainly relevant, but is deferred to later studies.

## Results

In this section, the turbulent flow around a three-dimensional Flettner rotor in an atmospheric boundary layer is presented. First, integral quantities, such as lift and drag coefficients, are discussed, highlighting differences compared to simplified potential flow theory predictions. Then, an overview of the instantaneous vortical structures illustrates the complexity of the flow. The statistical quantities are also used to characterise the cylinder wake and discuss the suppression of vortex shedding as the cylinder begins to spin.

### Aerodynamic loads

**Figure 2:**
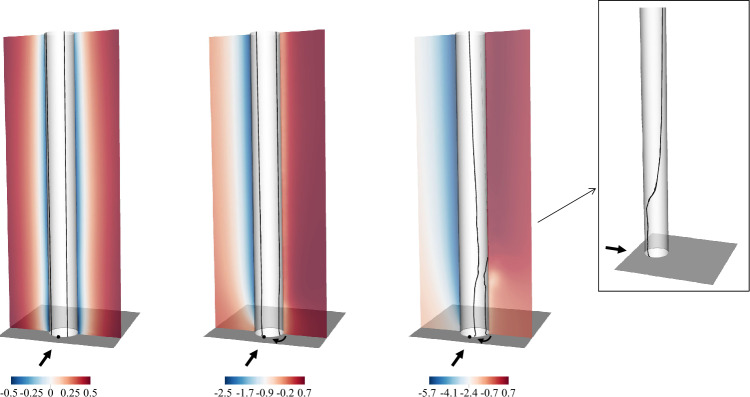
From left to right ($\alpha =0,1.5,3$), contours of the time-averaged pressure in the *yz* plane at $x=0$. The black arrows indicate the inflow and spinning velocities directions, whereas the point at the wall indicates the angle $\theta =180$°, measured clockwise from the *x*-axis. The pressure isolines $\overline{p}=0$ on the cylinder surface are indicated with solid dark lines. Note that only a small portion of the domain is shown: $x/D \in [-0.5,3]$, $z/D \in [-3,3]$.

The accurate prediction of aerodynamic loads in complex geometries, such as the Flettner rotor, is challenging. In preliminary design phases, the flow is usually modelled as ideal, with zero viscosity ($\nu =0$). In two-dimensional geometries, with the additional assumption of irrotational flows ($\varvec{\omega }= \mathbf {\nabla } \times \varvec{u}=0$), the flow can be described by a Laplace equation for the velocity potential ($\phi$): $\nabla ^2 \phi = 0$, supplemented by appropriate kinematic boundary conditions. In all irrotational flows, a velocity potential must exist, and they are commonly referred to as potential flows^19^. Due to linearity, different types of flows can be modelled using the superposition principle, where the flow solution results from a linear combination of elementary flows. The flow around a cylinder can made by a horizontal uniform stream in the streamwise direction combined with a dipole. This leads to the well-known d’Alembert’s paradox^19^ because the symmetric pressure distribution results in a net zero pressure force on the cylinder. A second discrepancy is related to the generation of lift forces when there is an asymmetric separation of the boundary layer, such as when rotation is introduced. This can be represented by incorporating another elementary solution, namely the irrotational vortex. In this way, low ($P_{-}$) and high ($P_{+}$) pressure regions are formed, giving rise to a lateral force (*lift*). Such principle, known as the Magnus effect, is commonly leveraged by Flettner rotors to harness wind energy from crosswinds and produce thrust. Despite the common application of potential theory to estimate lift force, significant disparities arise in real flow conditions. Viscous boundary layers form on solid surfaces, and the thickness of these boundary layers is essential due to the viscous diffusion of vorticity. Moreover, wake regions develop when boundary layers detach from the surface where they originated, creating a broader region of rotating flow^19^. Let us take a close look at how the aerodynamic loads vary in the real flow of our simulations.

Compared to potential flows, a first discrepancy appears in the pressure difference in the crossflow *yz* plane at $x=0$, see Fig. 2. Note that, in the rest of the manuscript, the normalised pressure is stated *negative* or *positive* with respect to the reference pressure ($P_\infty$). The black line indicates the location on the cylinder surface where the pressure becomes negative (i.e. the zero pressure contour), indicating the possible beginning of an adverse pressure gradient. Based on the potential flow described earlier, the predicted angle is approximately $\theta \approx 113$°(measured clockwise from the *x*-axis, as indicated here and throughout this document). It is important to note that this estimation is valid for two-dimensional irrotational flows. In contrast, the present configuration is three-dimensional and introduces additional complexities, including the velocity shear layer and interactions between the cylinder and the wall. Consequently, the pressure deficit zone differs significantly. For $\alpha =0$, the two points are symmetric with respect to the *x*-axis, as expected. As the cylinder starts to rotate, they shift by almost 70°, but the lines remain vertically straight. With further increases in spinning, the two points are significantly influenced by the velocity shear layer, resulting in varying boundary layer separations along the *y* direction. For a circular cylinder, as the spinning rate tends to infinity, the flow can be approximated as irrotational^34^. However, at lower rates of cylinder rotation, such as in this case, the decelerated boundary layer is unable to overcome the adverse pressure gradient behind the cylinder, likely resulting in separation. Nonetheless, even in the presence of separation, observed flow velocities are higher on the $P_{-}$ side of the cylinder, indicating the generation of lift force.

**Figure 3:**
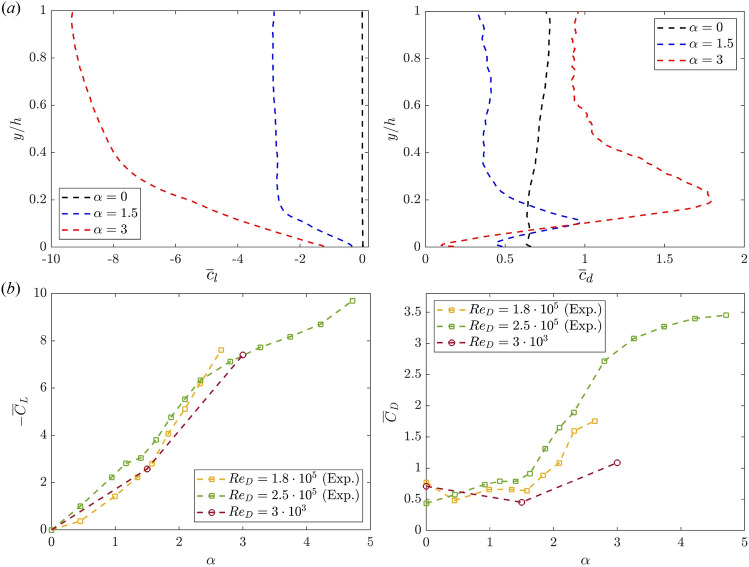
(**a**) The time-averaged (*left*) lift and (*right*) drag coefficients of the rotor as a function of the cylinder height (*y*/*h*). The rotation ratios $\alpha =0,1.5,3$ are indicated with dashed dark, blue and red lines respectively. (**b**) The time-averaged (*left*) lift and (*right*) drag coefficients integrated along the wall-normal direction as a function of $\alpha$. Experimental data by Bordogna et al.^11^ are also included.

The time-averaged vertical variation of the lift and drag coefficients are shown in Fig. 3a. The aerodynamic force is computed by time-averaging the surface integral of the pressure and viscous stress tensor: $\mathbf {F_a} = \int _S (-p \textbf{n} + \varvec{\tau } \textbf{n}) \hspace{1.1mm} \text {d} S$, where *S* is the cylinder surface, *p* is the pressure, $\textbf{n}$ is the vector normal to the cylinder surface and $\varvec{\tau }$ is the viscous stress tensor $\tau _{ij}=\mu (\partial u_i/\partial x_j+\partial u_j/\partial x_i)$. Following the previous work by Bordogna et al.^11^, the lift and drag components are normalised by $1/2 \rho U_{cl}^2 h D$.

As expected, the lift coefficient is zero for $\alpha =0$. When rotation is introduced, the pressure distribution gets asymmetric and the pressure difference across the cylinder results in a lift force. The lift increases with $\alpha$ and closely matches previous experiments at much higher Reynolds numbers^11^. However, the lift generated is not uniform along the vertical direction, especially for $y/h<0.3$ at $\alpha =3$, see Fig. 3a. The integrated value agrees well with previous measurements by Bordogna et al.^11^. For bluff bodies like cylinders, most of the drag arises from flow separation and wake formation, which can be unsteady or even turbulent, as observed here. Figure 3a illustrates the time-averaged vertical variation of $\overline{c}_d$. In the non-rotating case, the drag linearly increases in the vertical direction, with a slope of approximately $\approx 0.1 y$. Remarkably, for $\alpha >0$, a different trend is observed. In particular, a significant portion of the drag is generated close to the wall, where the impingement of the cylinder and the lift-up effect created by the shear layer combine to increase the resistance. A possible connection with the local adverse pressure gradient and the large-scale vortical motion of the wake presented below is also plausible and detailed in section "Large-scale motion at the cylinder base". In contrast to the lift, the drag coefficient is more affected by the Reynolds number. This explains a larger discrepancy with the experimental data by Bordogna et al.^11^ when the integral lift ($\overline{C}_L$) and drag ($\overline{C}_D$) coefficients are calculated, see Fig. 3b. However, we also reported a trend for which the $\overline{C}_D$ first decreases for lower rotation ratios ($\alpha =1.5$), and eventually increases ($\alpha =3$). This is related to the significant shift of the pressure deficit $P_{-}$ zone reported in Fig. 2. A similar trend was reported by Refs.^11,12^. Eventually, we could also estimate the viscous and pressure contribution to the drag. In all cases, the pressure component is dominant, contributing by more than $86\%$.

### Instantaneous vortical structures

**Figure 4:**
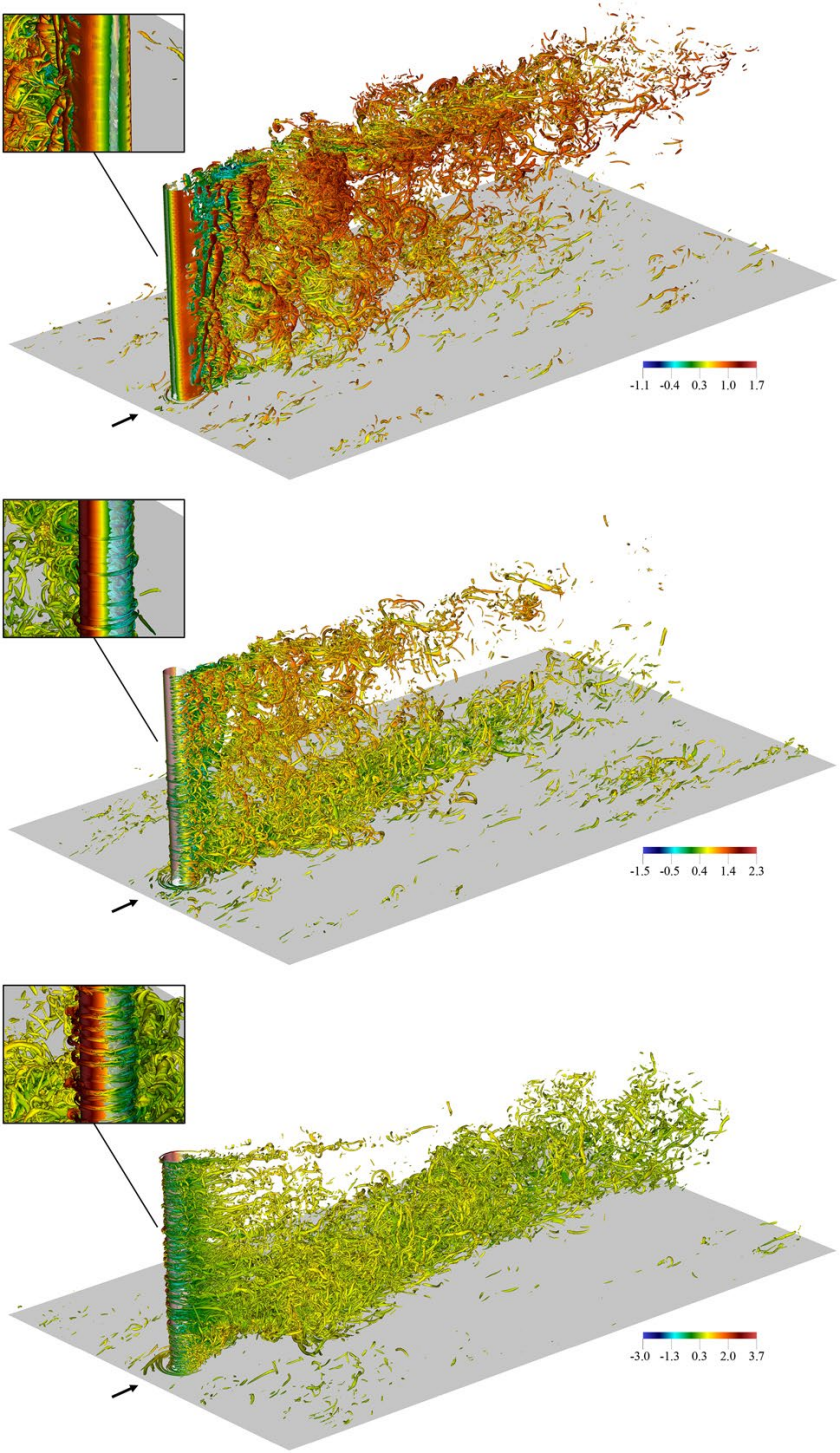
Instantaneous $\lambda _2$ visualisations ($\lambda _2=-100 \hspace{1mm} U_{cl}^2/D^2$) coloured with the instantaneous streamwise velocity in a portion of the domain for (*top*) $\alpha =0$, (*middle*) $\alpha =1.5$ and (*bottom*) $\alpha =3$.

In this section, we provide a general overview of the vortical structures that develop around the Flettner rotor. Although an unequivocal definition for the minimal turbulent unit as the vortex does not yet exist^35^, to identify organised and coherent flow structures, the $\lambda _2$-criterion proposed by Jeong and Hussain^36^ is used. This criterion is based on the second-largest eigenvalue, $\lambda _2$, of the symmetric tensor $\textbf{S}^2 + \mathbf {\Omega }^2$, where $\textbf{S}$ and $\mathbf {\Omega }$ represent the symmetric and antisymmetric components of the velocity gradient tensor $\nabla \textbf{u}$. For consistency, all $\lambda _2$ values are normalised by $U_{cl}^2/D^2$ from this point onward. The complexity of the flow and the exploratory nature of this study make the identification of vortical motions a non-trivial task. Furthermore, this configuration combines elements from various canonical flows, including a velocity shear layer, a rotating bluff body, and cylinder-wall interaction. Nonetheless, we can observe some noteworthy features and their evolution with the spinning ratio.

Figure 4 (*top*) illustrates the swirling regions around a non-rotating cylinder in a shear flow, identified by isocontours of negative $\lambda _2$. When a shear flow encounters a vertical cylinder, it gives rise to horseshoe vortices (H) in the junction region between the wall and the cylinder, clearly visible at the base of the cylinder. The horseshoe vortex, as defined, consists of a trio of interconnected vortices^37^. These vortices include a bound vortex that encircles the small cylinder and two trailing vortices extending from the bound vortex into the wake region. The number of Hs and their oscillation frequency can vary depending on the regime, significantly affecting the wall shear stress^38^. Therefore, gaining a comprehensive understanding of the turbulent H-system is crucial for comprehending, predicting, and controlling the local dynamics in front of cylinders. As also observed by Cao et al.^14^, the horseshoe vortex tends to move farther upstream as the turbulent boundary layer gets thicker. Furthermore, as the cylinder starts to rotate, the number of secondary vortices increases substantially since the flow is induced to wrap around the cylinder by the rotation, as shown in Fig. 4 (*middle, bottom*). For $\alpha >0$, the impingement effect of the cylinder propagates further upstream, leading to a larger stagnation region in front of the cylinder. This stagnation region corresponds to the area where the flow separates from the cylinder surface, resulting in the formation of more intricate horseshoe vortex structures.

The wake for $\alpha =0$ exhibits the usual vortex shedding, creating alternating swirling patterns in the wake. These vortices form on both sides of the cylinder and detach periodically, resulting in a shedding pattern. However, the presence of the boundary layer generates a vertically inclined Kármán vortex street, with a linear inclination. The vortex street deflection is related to the shear layer of the inflow. In the near wake, the vortices are organised into vertical stacks (inclined as well) and the transition location in the cylinder shear layer is not fixed across the span, as shown in the zoomed-in picture of Fig. 4 (*top*). The vertical non-uniform transition generates cells of the shear layer vortices that cause dislocations^39^, contributing to the development of wake three-dimensionality. The three-dimensional wake develops streamwise structures connecting the shed vortices, with both small-scale braids and large-scale helically twisted patterns downstream in the wake (a result of a mode B three-dimensional wake instability^40^). Similar patterns have been observed in the context of uniform^41^ and stepped^42,43^ cylinders. In the current case, they not only wrap in the streamwise direction but also develop vertically, carried by the velocity shear. As the cylinder starts to rotate, the wake complexity increases even further. For $\alpha =1.5$, vortex shedding is still observed, especially in the upper part of the cylinder. However, shedding is suppressed for $y/h \lessapprox 0.3$, as discussed later. Figure 4 (*middle*) provides evidence that the wake is divided into two vertical stacks, independent of the $\lambda _2$ value. The vertical separation occurs consistently at $y/h\approx 0.3$, where vortex shedding is suppressed. Further details about the vortex shedding as a function of *y* are provided in Sect. 3.4.

Close to the cylinder surface, deep within its boundary layer, we observe another noteworthy aspect of the flow. The middle zoomed-in picture of Fig. 4 shows small vortical structures wrapping around the rotor. These structures, with axes oriented azimuthally, are induced by the cylinder rotation and resemble counter-rotating toroidal vortices in Taylor–Couette flow, called Taylor vortices. These secondary flows are particularly prominent in the lower part of the cylinder for $\alpha =1.5$. As the spinning ratio ($\alpha$) increases, they have a more regular pattern in the vertical direction but also exhibit instabilities. The velocity shear layer around the cylinder steepens due to the higher spinning velocity ($U_{sp}$), and the wrapping vortices result in hairpin-like vortical motions on the cylinder surface. As previously discussed in stepped geometries^43^, hairpins only appear when the cylinder shear layer becomes unstable, which, in this case, is dependent on the spinning ratio. These flow characteristics can only be accurately described and analysed through high-fidelity DNS, which captures small-scale dynamics. Thus, neglecting these structures, the flow instabilities around the rotor could not be properly depicted.

### Large-scale motion at the cylinder base

**Figure 5:**
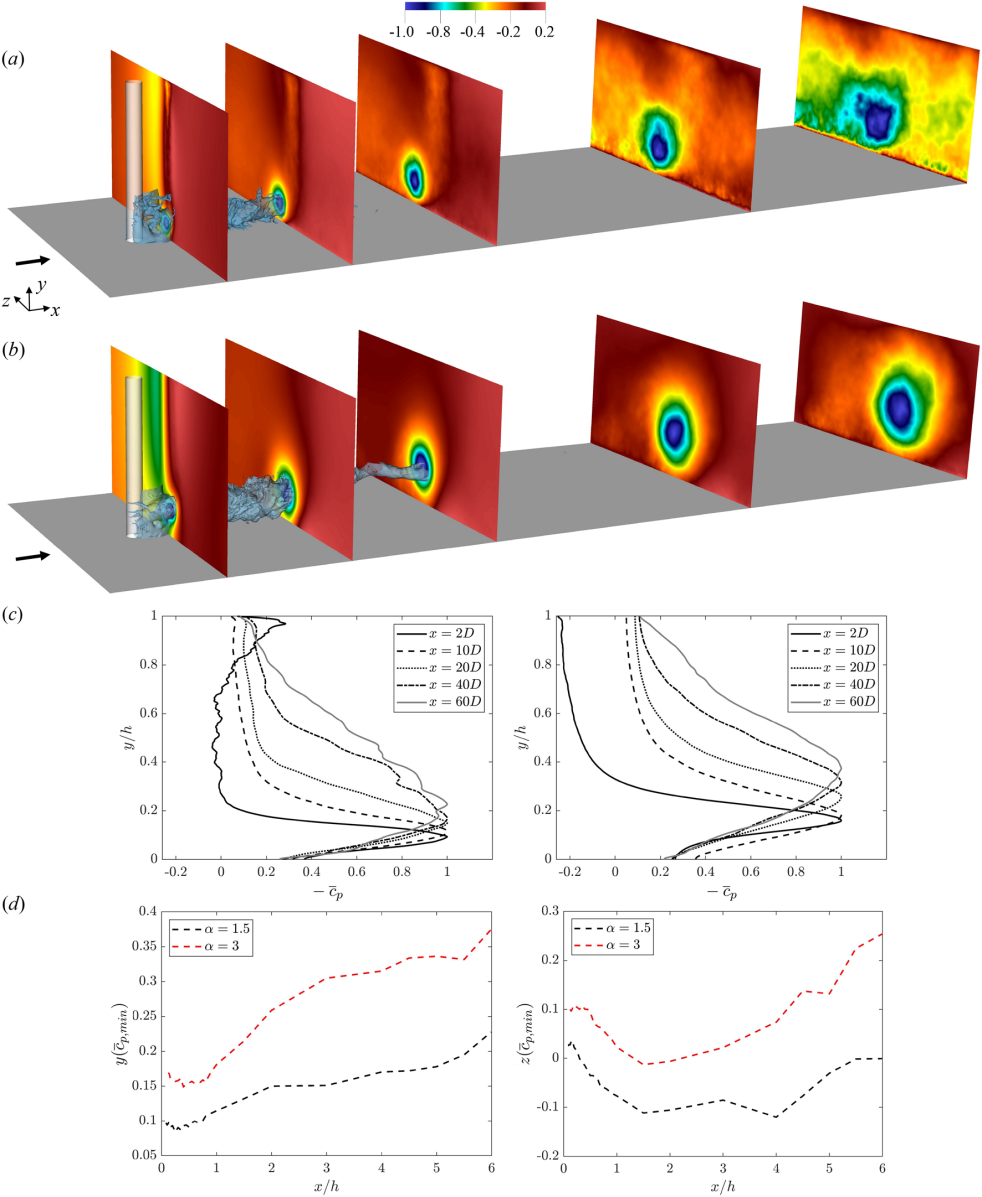
(**a**,**b**) The time-averaged (local) pressure in *yz* planes at $x/D=2,10,20,40,60$ for $\alpha =1.5$ and $\alpha =3$. In light blue, the instantaneous low-pressure isosurface shows the large-scale motion developing at the basis of the cylinder. A small portion of the domain is shown. (**c**) The vertical variation of the time-averaged pressure (locally normalised with its maximum) in the same planes displayed above. The pressure profile is extracted at the *z* location of the pressure minimum. (**d**) The streamwise variation of the pressure minimum in the (*left*) vertical and (*right*) spanwise direction.

As the cylinder starts to rotate, at the base of the cylinder a large-scale streamwise vortical motion develops. The wrapping motion is connected to the wake deflection at the wall, as shown in Fig. 4. In fact, Fig. 4 (*middle, bottom*) only show the small-scale coherent motions through $\lambda _2$ structures, but a large-scale coherent pattern also appears. This was shown already visible in the animation by Massaro et al.^44^, where preliminary results were presented for the highest rotation rate, $\alpha =3$. The signature of this structure is clearly evident in the time-averaged statistics. Capturing this prominent large-scale feature is crucial for accurate drag estimation and for the future development of control strategies to minimise its impact. In this study, we further characterise its evolution with respect to $\alpha$, analyse its spatial distribution statistically, and discuss its origin. Figure 5a and b show the contours of the time-averaged pressure in *xz* planes at various locations in the wake for $\alpha =1.5$ and $\alpha =3$. The pressure minimum is used to track the position of the large-scale motion developing at the base of the rotor, and the consequent wake deflection. The minimal dissipation of spectral element method (see Sect. 5) allows for tracking its evolution for several tens of diameters downstream in the wake. It is worth noting that Fig. 5 shows a portion of the domain, up to $x/D=60$.

The ambient boundary-layer flow is essentially shear-driven, which is accomplished in a channel setting by a small negative pressure gradient. However, the rotation of the cylinder induces low and high-pressure regions on the cylinder sides, as sketched in Fig. 1a and shown in Fig. 2. Consequently, the turbulent boundary layer in which the cylinder is immersed experiences a localised increase in pressure, creating an adverse pressure gradient (APG) area on the $P_{+}$ side. This is also demonstrated by the negative (time-averaged) wall shear stress in the same region (necessary, but not sufficient, condition for separation). The local APG leads to flow separation on the front part of the cylinder ($P_{+}$ side). The lifting-up motion, sustained by the incoming flow and enhanced by the cylinder spinning action, generates a large-scale vortical structure that travels downstream, as depicted in Massaro et al.^44^. The explanation for the vortex formation provided here is based on both instantaneous and statistical data. Furthermore, when flow separation occurs, aerodynamic performance usually deteriorates due to an increase in drag. This is precisely what occurred with the aerodynamic coefficient discussed in Sect. 3.1. In Fig. 3a (*right*), a significant increase in the cylinder drag is evident at a similar height where the vortex is observed.

The vortical motion progressively diffuses downstream, as shown by the normalised pressure variation in the crossflow *xz* planes at $x/D=2, 10, 20, 40,$ and 60. Also, as $\alpha$ increases, the thickness of the vortical swirling increases. The growth is initially limited for $x/D<20$, but it is significant as the vortex is advected further downstream. To quantitatively measure its extension, let us define the thickness of the vortex as the height where the ambient value of $\overline{c}_p$ is attained, assuming that the vortex is always attached to the wall. For $\alpha =3$, at $x/D=60$, the vortex size is comparable to half of the cylinder height (*h*). In contrast, for $\alpha =1.5$, it is smaller, around 0.2*h* (Fig. 5c). The vertical location of the swirling structure is also interesting. For $\alpha =1.5$, the pressure minimum remains initially relatively close to the wall, reaching at most a height of $y/h\approx 0.2$ at $x/D=60$. In contrast, for $\alpha =3$, the vortex is more lifted upstream, with the pressure minimum location at $y/h\approx 0.37$ at $x/D=60$. Likely this is due to the larger separation occurring as $\alpha$ increases. For $\alpha =1.5$ and $\alpha =3$, the pressure minimum is at $y/h\approx 0.1$ and $y/h \approx 0.2$, in the near wake. Since the cylinder is immersed in a vertical velocity shear layer, the two large-scale vortical structures have two entirely distinct vertical advection patterns.

Finally, let us consider the spanwise location of this structure (Fig. 5d, *right*). In both cases, the vortex originates at positive spanwise positions on the cylinder, corresponding to the $P_{+}$ side, and is subsequently deflected downwards. This behaviour is explained by the lower-pressure field at positive spanwise locations ($P_{-}$ side) generated by the cylinder rotation, leading to its *z*-deflection. For $\alpha =1.5$, it even crosses the $z=0$ plane. However, in both cases, after the initial deflection related to the depression generated by the rotation, it tends to align with the main flow direction. For $\alpha =1.5$, it is recovered after 60*D* with a location around $z/D=-0.05$. For $\alpha =3$, it is not fully recovered even after 100*D*, and it is significantly shifted in the spanwise direction, with a pressure minimum around $z/D\approx 0.25$.

### Vortex shedding suppression

**Figure 6:**
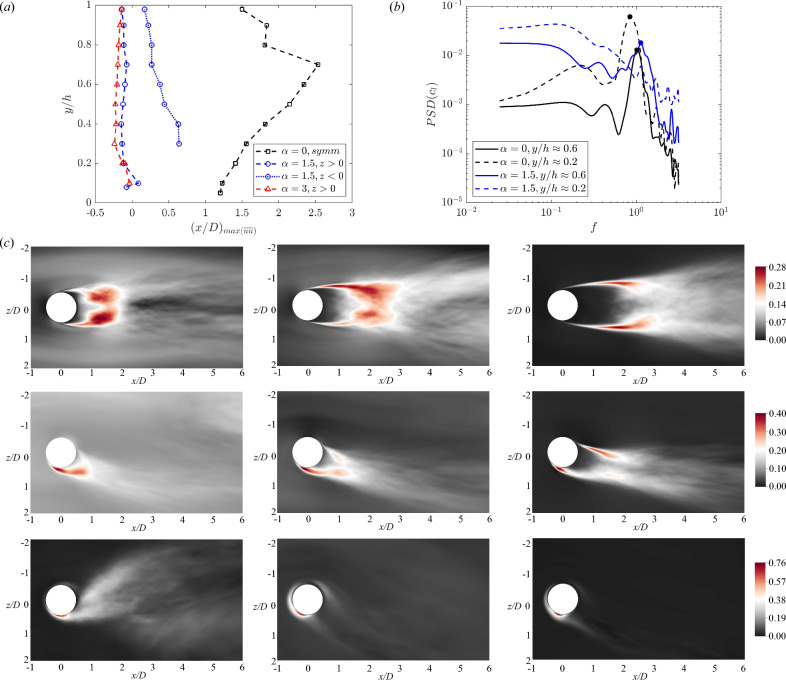
(**a**) Vertical variation of the streamwise location of the vortex formation length ($(x/D)_{max(\overline{uu})}$). (**b**) Welch’s power spectral density (PSD) is estimated for the sectional lift coefficients ($c_l$). The dots indicate the vortex shedding at the frequency $f \approx 1$. (**c**) The time-averaged streamwise component of the Reynolds stress tensor in the *xz* planes at (*top*) $\alpha =0$, (*middle*) $\alpha =1.5$ and (*bottom*) $\alpha =3$. From left to right, different wall-normal distances are considered: $y/h=0.1,0.6,0.9$. The cylinder rotation is clockwise.

As previously depicted by the instantaneous flow visualisations, vortex shedding is suppressed as $\alpha$ increases. The suppression of vortex shedding in rotating cylinders remains a subject of debate. When considering a homogeneous inflow, it is observed that for $\alpha \ge 1.5$, the Kármán activity in the wake deteriorates and ultimately gets suppressed. However, it is crucial to account for the initial transient to prevent drawing erroneous conclusions related to transient behaviours, as discussed by Mittal and Kumar^15^. In the current study, the first 20 flow-through units, based on $U_{cl}$ and *h*, are discarded. This corresponds to more than 60 vortex-shedding periods. Previous studies reported that this initial transient phase is adequately long for the $\alpha$ values under investigation^15^. Note that, in contrast to homogeneous flows, the vertical velocity shear layer increases the flow complexity since the suppression can vary with *y*, as discussed below.

The statistical evidence of vortex shedding is quantified by using the vortex formation length, which is defined as the distance between consecutive vortex shedding points. Among the different definitions available in the literature, in this study, the vortex formation length is defined using the location of the maximum time-averaged streamwise velocity fluctuation $\overline{uu}$, following the previous works^42,43,45,46^. In the near wake, two peaks are expected to occur when the Kármán vortex sheet is formed. Indeed, for $\alpha =0$, the vortex formation length displays a symmetrical double peak with respect to the $z=0$ plane. The peaks progressively approach the cylinder for $y>0$, as they are stretched by the velocity shear layer (Fig. 6c). This value aligns with data available in the literature, indicating a formation length of approximately 2–3 cylinder diameters (*D*). A reduction is observed for $y \gtrapprox 0.75$. This decrease is not related to a change in the vortex shedding pattern, but rather to the migration of the $\overline{uu}$ peak. The vertical velocity gradient creates an elongated recirculation region in the near wake, and the peak is located in the braid shear layer^40^. These braids are responsible for the instability mechanism that generates mode B streamwise vortices. The situation is different when the cylinder begins to rotate, and the symmetry is lost. In the context of a homogeneous inflow, a value of $\alpha =1.5$ was previously reported as the limit for sustaining vortex shedding^15^. However, when the cylinder is placed in an open turbulent channel, the situation changes significantly. Indeed, a composite behaviour is observed, where vortex shedding is only suppressed at specific *y* locations. Notably, the time-averaged streamwise component of the Reynolds stress tensor exhibits a single positive peak for $y/h<0.3$, as shown in Fig. 6c. The peak is situated on the $P_{+}$ side, close to the large-scale motion formation. At this intermediate $\alpha$, as we move vertically, the influence of rotation is highly moderated by the velocity shear layer, and vortex shedding resumes for $y/h>0.3$. Specifically, for a given $y_2>y_1$, we have $\overline{U}(y_2)>\overline{U}(y_1)$, but the spinning contribution $U_{sp}$ remains constant with respect to *y*. Thus, it is reasonable to expect that vortex shedding can resume on both sides, as indicated by the time-averaged statistics. Note that to identify the reappearance of vortex shedding on both sides, we considered a second peak with a value of at least 50% of the maximum $\overline{uu}$. Eventually, for $\alpha =3$, vortex shedding is suppressed at every *y* station, as clearly illustrated in Fig. 6c. Vortices are only shed for $z>0$, and the formation length is very similar to that of $\alpha =1.5$. In both cases, the location $(x/D)_{max(\overline{uu})}$ is in proximity to the cylinder, typically between $-0.24D$ and 0.08*D*, with the vortex actually detaching at an angle of approximately $\theta \approx 110$°clockwise from the *x*-axis. These observations are corroborated by the time series of the aerodynamic loads, especially the sectional lift coefficient ($c_l$). The power spectral density (PSD) computed by the Welch method, shows a distinct peak connected to the vortex shedding at $f\approx 1$ for $\alpha =0$, see Fig. 6b. The peak is still visible for $\alpha =1.5$ in the upper part of the cylinder, at $y/h=0.6$, but it disappears closer to the wall where the vortex shedding is suppressed. The spectra for $\alpha =3$ are similar to the spectrum for $\alpha =1.5$ at $y/h\approx 0.2$, as shown in Fig. 6b, with no distinct vortex shedding peak.

### Effect of rotation on the near wake velocity profiles

**Figure 7:**
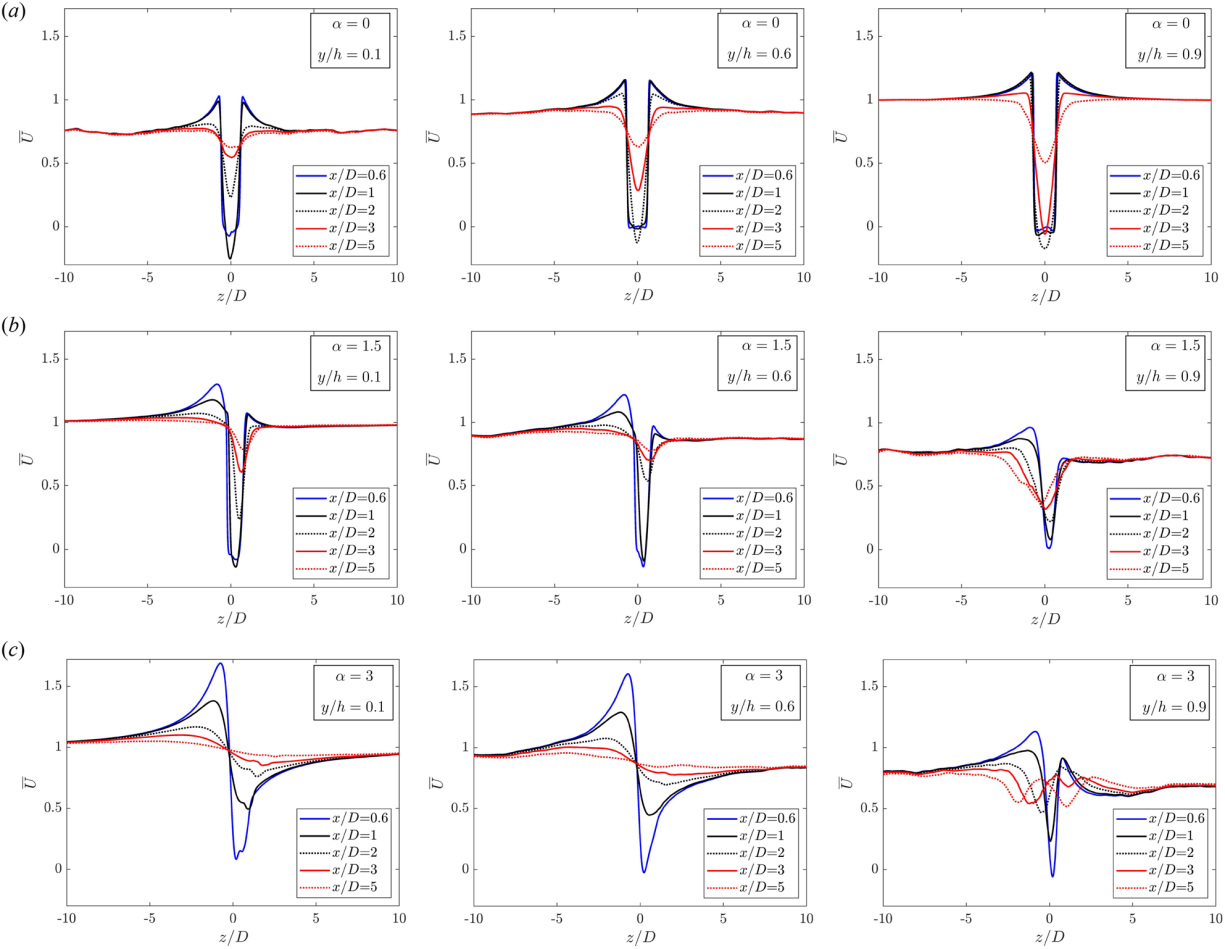
(**a**–**c**) The time-averaged streamwise velocity profile in the near cylinder wake for different rotation rates: $\alpha =0,1.5,3$. From left to right, various wall-normal distances $y/h=0.1,0.6,0.9$.

Finally, the impact of rotation on the near wake of the cylinder is characterised by analysing the mean velocity profile ($\overline{U}$) at various streamwise locations. The wake of the bluff body in a vertically homogeneous flow has been extensively examined in the past. Self-similar solutions emerge when the independent spatial and temporal variables can be combined and scaled in the downstream direction. The velocity profile of the wake, akin to a jet, approaches a self-similar shape not far from the object. However, in more complex geometries, such as the one under investigation, this behaviour changes. The flow is not only non-homogeneous in the vertical direction but also rotates with respect to the *y* axis.

Let us begin with the non-rotating case (Fig. 7a). Along the cylinder (*central* and *right* pictures), the negative velocity deficit ($\overline{U}<0$) is more pronounced at a location of $x/D=2$, rather than very close to the cylinder ($x/D\le 1$), where the streamwise velocity remains positive. This is potentially explained by the variation in the vortex formation length described above. As the rotation initiates, the rotational effect becomes evident in the wake velocity deficit as well (see Fig. 7b and c). It is important to note that farther away from the wall (*right*), the velocity gap is considerably smaller due to the larger streamwise velocity inflow. However, at these higher vertical locations, it takes a much longer streamwise distance to fully recover, as indicated by the red lines at the farthest downstream distance. The velocity asymmetry as a function of *y* is also noteworthy. Specifically, for $\alpha =1.5$, at $y/h=0.1$ and 0.6 (Fig. 7b, *left* and *centre*), the increase in velocity on the $P_-$ side is more substantial than the deficit on the $P_+$ side. Consequently, at a location 5*D* downstream of the wake, the positive velocity surplus is fully recovered, unlike the negative deficit (red lines). Finally, at the highest spinning rate, an almost symmetric wake with positive/negative deficits is observed (Fig. 7c). At the topmost vertical location (*right*), a double change in curvature becomes evident at positions $x/D=3$ and 5 (red lines). This phenomenon is likely associated with the more significant depression induced by spinning, which draws mass flow from the external region, resulting in a central increase in the streamwise velocity component. This central region recovers more rapidly than the flow farther away from the horizontal axis ($x=0$).

## Discussion and conclusions

We perform detailed direct numerical simulations of the three-dimensional turbulent flow around a Flettner rotor, i.e. a rotating cylinder in an atmospheric boundary layer. The work investigates various aspects of the flow, including the forces on the cylinder and the vortical structures arising from the cylinder, quantifying the effect of rotation for three different spinning ratios $\alpha =0,1.5,3$ on the cylinder wake.

In such a realistic flow configuration, as opposed to the typical simplified potential flow models, the aerodynamic loads, based on both viscous and pressure forces, may be estimated accurately. We quantify the locations where the pressure changes sign along the cylinder, i.e. where $\overline{p}<0$ and separation might begin. For $\alpha =3$ the separation line ($\overline{p}=0$) shows a significant fluctuation along the vertical direction, leading to a substantial shift of the wake entertainment vertically. This is also reflected in the vertical (*y*) variation of the lift and drag coefficients. The time-averaged lift coefficient increases with $\alpha$, and the upper part of the cylinder makes a significant contribution to the lift. In contrast, the time-averaged drag coefficient shows that a substantial portion of the drag, especially pressure drag, originates from the near-wall region when the cylinder starts to rotate. This is likely promoted by the large-scale motion observed at the base of the cylinder. Both instantaneous and statistical data reveal the presence of a large-scale structure at the cylinder base, characterised by a streamwise wrapping of the flow near the wall, which significantly alters the wake. We conclude that it originates from the adverse pressure gradient (APG) present on the $P_{+}$ side of the cylinder. The APG is generated by the spinning motion of the cylinder, which explains the occurrence of such motion only for $\alpha >0$. Examining the variation of the pressure minimum in *yz* planes allows us to characterise the size of this vortical motion, which has been reported in this study for the first time. The structure persists for a streamwise distance exceeding 60*D*. This finding holds particular relevance in marine applications, as it can substantially affect following ships. Furthermore, attempting to control the wake and reduce the drag (and consequently fuel consumption), this result provides valuable insights on where to apply control techniques. Another intriguing result pertains to the suppression of vortex shedding. This phenomenon continues to be a subject of debate in the literature, and its examination in complex geometries (such as the present one involving a velocity shear layer and interactions with the wall) is of utmost importance. In the case of intermediate $\alpha =1.5$, the suppression of vortex shedding is only partial. For instance, vortices continue to be shed at $y/h>0.3$, a fact that can be quantitatively measured by the vortex formation length. However, for $\alpha =3$, vortex shedding is entirely suppressed along the entire length of the cylinder. This observation is crucial since the vortex shedding directly impacts the frequency of oscillating loads on the cylinder. Factors like fatigue are greatly influenced by the presence or absence of such oscillating loads, and non-uniform vertical loading has the potential to considerably affect the structural stability of the rotor itself.

This study presents the direct numerical simulation of turbulent flow around a Flettner rotor, considering various rotation rates. The primary objective of this research is to unveil the underlying mechanisms of this specific flow configuration. Flettner rotor technology holds huge significance in both current and future marine propulsion systems, offering a sustainable alternative in an industry responsible for a significant portion of total transport emissions. Additionally, our simulation campaign is conducted within a state-of-the-art numerical framework that leverages graphics accelerators. The graphics processing units (GPUs) have been demonstrated to yield substantial energy savings in comparison to traditional architectures^47^, aligning with sustainability goals in the realm of computational fluid dynamics (CFD) research. Moreover, they enable the execution of large-scale simulations, such as the one presented here, which were previously unattainable. In the future, it would be intriguing to delve deeper into the parameter space defined by rotation rate ($\alpha$) and Reynolds number ($\textrm{Re}$). This latter is still far from realistic applications as this study represents an exploratory work, striving to understand fundamental mechanisms in such a complex configuration. Indeed, it was possible to unveil mechanisms that are expected to persist as *Re* increases. As resolved simulations at realistic Reynolds numbers are still infeasible, we deemed it more important to resolve the near-wall effects (including separation, laminarisation, etc.). Investigating the impact of the free-end surface or exploring tandem configurations could also yield valuable insights, contributing to a more comprehensive understanding of real-world applications.

Eventually, an unexplored phenomenon in the literature, with potentially significant implications for Flettner rotor operation, is the so-called *inverse Magnus effect*. In the study of flow around a rotating sphere, an intriguing observation emerges: within a specific range of rotation speeds, the lift direction alters^32^. For the Flettner rotor, this would lead to a drag force rather than thrust. Kim et al.^32^’s experimental findings suggest an explanation related to the drag crisis and sphere boundary layer transition. To explore this phenomenon further, a high-fidelity DNS of a rotating cylinder at a very high Reynolds number ($Re_D \approx 100,000-200,000$) is essential to capture the cylinder boundary layer transition, and subsequently, the potential inversion of force direction while rotating. In the context of the Flettner rotor, the possibility of having direct and inverse Magnus effects at different heights is even more intriguing.

## Methods

Direct numerical simulations are conducted using the open-source solver Neko, a modern framework for computational fluid dynamics (CFD)^48^. Neko has its origins in Nek5000^49^, a CFD solver known for its favourable numerical properties, such as minimal dissipation, and its scalability on multicore processors. Neko is a newly developed code that leverages object-oriented Fortran to manage memory allocation and enable multi-tier abstractions of the solver stack. It is designed to support various hardware backends, including general-purpose processors, accelerators, vector processors^50^, and, to some extent, Field-Programmable Gate Array (FPGA) support^51^. Although the project is still in its early stages, Neko has been already selected as a finalist for the prestigious ACM Gordon Bell Prize in 2023^52^.

The solver integrates the incompressible, non-dimensional, Navier–Stokes equations

1 $$\begin{aligned} \left\{ \begin{array}{ll} \displaystyle \dfrac{\partial \varvec{u}}{\partial t} + \left( \varvec{u}\cdot \nabla \right) \varvec{u}= - \nabla p + \dfrac{1}{\textrm{Re}_{cl}} \nabla ^2 \varvec{u}, \\ \nabla \cdot \varvec{u}= 0, \end{array} \right. \end{aligned}$$

where $\varvec{u}$ is the non-dimensional velocity, *p* is the non-dimensional pressure, and $\textrm{Re}_{cl}=U_{cl}h/\nu$ is the Reynolds number based on the cylinder height *h*, the centerline velocity $U_{cl}$ and the kinematic viscosity $\nu$. The set of equations is incomplete without proper initial and boundary conditions. At the inflow ($x/D=-50$) the Dirichlet boundary condition (BC) prescribes an open turbulent channel velocity profile with the power-law $u_x/U_{cl}=(y/h)^{1/7}$. A similar condition is used at the initial time. The outflow at $x/D=120$ consists of natural boundary condition $(-p \textbf{I} + \nu \nabla \varvec{u})\cdot \textbf{n} = 0$. We consider an infinitely long cylinder in the *y* direction, thus symmetric Robin (mixed) boundary conditions are prescribed at the top surface ($\varvec{u}\cdot \textbf{n} = 0$ with $(\nabla \varvec{u}\cdot \textbf{t}) \cdot \textbf{n}= 0$), where $\textbf{t}$ is the tangential vector. For the spanwise boundaries, mixed conditions are prescribed to allow mass-flow transpiration. These are similar to an open boundary, but prescribing zero velocity increment in non-normal directions and avoiding adverse flow effects^53^. On the rotor, a Dirichlet boundary condition (BC) is employed, enforcing wall impermeability and setting a rotational velocity $u_{\theta } = U_{sp} = \alpha U_{cl}$ with $\alpha = 0, 1.5, 3$, while $u_{\rho } = 0$. In this context, the BC is expressed in cylindrical coordinates, with $u_{\theta }$ and $u_{\rho }$ representing velocities in the azimuthal and radial directions, respectively. To avoid incompatible conditions at the base of the cylinder, the spinning velocity is smoothed as a function of *y*

2 $$\left\{ {\begin{array}{*{20}l} {s(y = 0) = 0,} \hfill \\ {s(0 < y < \delta ) = U_{{sp}} /(1 + e^{{1/(q - 1) + 1/q}} ),} \hfill \\ {s(y \ge \delta ) = U_{{sp}} } \hfill \\ \end{array} } \right.$$

where $q=y/\delta$ and $\delta =0.02h$. To reduce the streamwise extent of the computational domain and sustain turbulence, the laminar-turbulent transition is initiated via the boundary layer tripping introduced by Schlatter and Örlü^54^ and available in the KTH framework^55^. It consists of a stochastic forcing term which is added in a region centred along a user-defined line at $x_0/D=-45$ and it extends from $z/D=-10$ to $z/D=10$. The implementation follows Schlatter and Örlü^54^, using a weak random volume force that acts in the *y* direction. This consists of a forcing term in the Navier–Stokes equation with a continuous first-order derivative in time that generates a noise with a uniform distribution over all frequencies lower than the specified cutoff frequency. For further details, please refer to Karp et al.^47^.

For the numerical discretisation of the Eq. (1), Neko employs a spectral element method (SEM) which guarantees minimal dissipation and dispersion, high accuracy and nearly local exponential convergence. To further enhance computational efficiency, a matrix-free approach is implemented in the code, leveraging optimised tensor operations and minimising communication costs. The SEM decomposes the domain into non-overlapping hexahedral elements where the solution is locally represented with polynomial basis functions. These basis functions are constructed using Legendre polynomials of degree *N* and are interpolated on the Gauss–Lobatto–Legendre (GLL) points. The same function space, *i.e* the same polynomial degree (*N*), is used for the pressure. This corresponds to the $P_{N}-P_{N}$ formulation^56^ and the polynomial order is $N=7$^57^. We employed established mesh criteria from the literature to guarantee an adequate resolution in all directions and a mesh convergence study was conducted^58^. Eventually, the time integration is achieved through a standard third-order backward differentiation scheme for the linear terms and a third-order extrapolation scheme for the nonlinear terms. For capturing the highest temporal frequencies, a time step of $\Delta t \hspace{0.5mm} U_{cl}/ h = 2\times 10^{-5}, 1.4\times 10^{-5}$ and $1\times 10^{-5}$ is used for $\alpha =0, 1.5$ and 3 respectively. They correspond to a Courant–Friedrichs–Lewy (CFL) number around $\approx 0.4$. For the statistics collection, the flow is sampled at each time step and the first 20 flow-through units, based on $U_{cl}$ and *h* are discarded. This corresponds to more than 60 vortex-shedding periods. As observed in previous studies^15^, and confirmed by our observations, this interval is sufficiently long to get rid of the transient. The statistics are collected for over 120 shedding periods, and statistical convergence is ensured. The numerical setup has been extensively validated for the flow around a homogeneous cylinder at $Re_D=3900$^59^, as well as for other flow cases^48,60^.

## Data Availability

All data needed to evaluate the conclusions are present in the paper. Additional data related to this work are available upon reasonable request to the authors.
